# Impact of early diagnosis of overt diabetes on pregnancy outcomes: a retrospective cohort study among Brazilian women

**DOI:** 10.20945/2359-4292-2026-0032

**Published:** 2026-04-01

**Authors:** Angela J. Reichelt, Maria Amélia A. Campos, Vânia N. Hirakata, Maria Lúcia Oppermann

**Affiliations:** 1 Serviço de Endocrinologia e Metabologia, Hospital de Clínicas de Porto Alegre; Programa de Pós-graduação em Ciências Médicas: Endocrinologia, Universidade Federal do Rio Grande do Sul, Porto Alegre, RS, Brasil; 2 Serviço de Endocrinologia, Hospital Nossa Senhora da Conceição, Porto Alegre, RS, Brasil; 3 Unidade de Bioestatística e Análise de Dados, Hospital de Clínicas de Porto Alegre; Programa de Pós-graduação em Cardiologia e Ciências Cardiovasculares, Universidade Federal do Rio Grande do Sul, Porto Alegre, RS, Brasil; 4 Serviço de Ginecologia e Obstetrícia, Hospital de Clínicas de Porto Alegre; Faculdade de Medicina, Universidade Federal do Rio Grande do Sul, Porto Alegre, RS, Brasil

**Keywords:** Type 2 diabetes, overt diabetes, diabetes in pregnancy, pregnancy outcomes

## Abstract

**Objective:**

Early diagnosis of overt diabetes has not been extensively explored. We compared pregnancy outcomes of women diagnosed with overt diabetes in the first trimester (up to the 13 completed weeks) to those with a later diagnosis.

**Subjects and methods:**

We evaluated women with overt diabetes and a type 2 diabetes phenotype. Pregnancy evolution (gestational weight gain, HbA1c, inpatient glycemic control) and outcomes (preeclampsia, neonatal hypoglycemia, and admission to the neonatal intensive care unit) were compared using multivariable analyses (linear regression or Poisson regression with robust variances). All models computed the early diagnosis and other specific clinically relevant outcome-related variables. Results are b (linear coefficient) or adjusted relative risk (RR) with 95% CI.

**Results:**

Of the 217 study participants, 127 (58.5%) were diagnosed in the first trimester (early diagnosis group); those with a late diagnosis presented a higher 3rd-trimester HbA1c (n = 169; b = 1.29; 0.04; 0.55, p = 0.026). Maternal inpatient care was associated with later diagnosis (n = 195; RR 1.29; 1.02; 1.63, p = 0.033) and higher initial HbA1c (n = 195; RR 1.25; 1.16; 1.35, p < 0.001). Aside from a better maternal metabolic control, early diagnosis of overt diabetes did not impact any other pregnancy outcome.

**Conclusions:**

In the present cohort of women with overt diabetes, diagnosis before the 14 gestational week modestly improved a few maternal metabolic aspects that did not translate into better pregnancy outcomes.

## INTRODUCTION

Overt diabetes is hyperglycemia that meets the diagnostic criteria for diabetes and is first detected at any time during pregnancy ^(1)^. Any case of diabetes can thus present as overt diabetes, probably representing a previously undiagnosed hyperglycemia, associated with the global rise in obesity and type 2 diabetes, now affecting women at progressively younger ages ^(2)^. Like gestational diabetes mellitus (GDM), overt diabetes can resolve after pregnancy, since only ~34% of women presented postpartum hyperglycemia in a meta-analysis ^(3)^; those diagnosed in the first trimester exhibited a higher prevalence of postpartum hyperglycemia ^(4)^.

During normal pregnancy, the pattern of glucose and insulin interrelation undergoes adaptive changes ^(5)^. Beginning at 12 to 14 gestational weeks, insulin sensitivity decreases, followed by an increase in insulin resistance with advancing gestation, which has been associated with placental hormone production ^(5)^. In pregnant women with type 2 diabetes, preexisting insulin resistance antagonizes the compensatory effect of the increased insulin sensitivity ^(6)^, unveiling hyperglycemia already in the early first trimester. For many authors, these findings at early pregnancy extend to mid-pregnancy, ~20 weeks of gestation ^(7)^. Since these metabolic alterations begin early in pregnancy, we chose the 14th gestational week, which represents the end of the first trimester in the Brazilian prenatal chart ^(8)^, as the cutoff point to divide our sample into early and late diagnosis groups.

Emerging evidence suggests that women with overt diabetes exhibit prevalence and risk of adverse perinatal outcomes comparable to those with pregestational type 2 diabetes ^(7,9)^, and generally greater than those associated with GDM ^(10)^. Early diagnosis and treatment of GDM, particularly before the 20th week, were associated with modest improvements in selected outcomes, including reduced incidence of neonatal respiratory distress, without affecting most outcomes ^(11)^. Few investigations have specifically addressed the impact of early versus late diagnosis of overt diabetes on pregnancy outcomes ^(7,12,13)^. Although overt diabetes should be identified by universal screening in the first trimester, as recommended by the World Health Organization ^(1)^, many cases are detected only later, when GDM screening traditionally occurs ^(14,15)^. Notably, an early diagnosis did not always translate into immediate treatment ^(10)^.

This study compared pregnancy outcomes according to the timing of overt diabetes diagnosis in a cohort of Brazilian women.

## SUBJECTS AND METHODS

### Participants and ethics statement

A secondary analysis was conducted using a retrospective cohort of participants with overt diabetes identified among 646 women with a clinical phenotype of type 2 diabetes ^(9)^. All women were enrolled between 2015 and 2021 at the two busiest public specialized prenatal care units in the Sistema Único de Saúde (SUS, the Brazilian public health system) in southern Brazil. The ethics committee of both hospitals approved the study protocol on July 28, 2016 (project number 2016-0331, *Hospital de Clínicas de Porto Alegre*). The study was registered in Plataforma Brasil under the national ethics appreciation certificate (CAAE) number 57365016.3.0000.5327, and the authors signed a data use agreement to ensure data confidentiality.

### Study variables

Overt diabetes was diagnosed at any time during pregnancy as per the 2013 World Health Organization guideline (fasting plasma ≥ 126 mg/dL or 2 h glucose after a 75 g load ≥ 200 mg/dL) ^(1)^ and/or according to the American Diabetes Association recommendation of glycated hemoglobin (HbA1c) ≥ 6.5% ^(16)^.

Since working with a complete dataset, we did not calculate the sample size; instead, we calculated the study power (type II error) for the main pregnancy outcomes. Target sample size and study power were calculated using the PSS-Health tool ^(17)^. We set a more conservative relative risk (RR) of 2.0, based on the relative risk of delivering large for gestational age babies (RR = 2.3) described by Jayasinghe and cols. ^(12)^ in a study with 58 participants, which compared risks between overt diabetes diagnosed in the first trimester to the other trimesters.

Data were retrieved from the electronic medical records. Demographic characteristics included age, schooling years, and skin color. Presence of diabetes complications (retinopathy, nephropathy, and/or neuropathy) and other baseline information, such as family history of diabetes or hypertension, smoking, chronic hypertension, prior gestational diabetes or macrosomia, and maternal admission for glycemic control, were collected as recorded in the hospital charts. Pre-pregnancy weight was self-reported, and height was measured at the first prenatal appointment. Weight was measured at each visit. We calculated the pregestational body mass index (BMI) as the informed pregestational weight in kilograms divided by the square of height, in meters, classifying women as normal pregestational BMI (BMI < 25.0 kg/m^2)^, overweight (BMI ≥ 25.0 kg/m^2^ to BMI < 30.0 kg/m^2)^, or obesity (BMI ≥ 30 kg/m^2)^ ^(18)^. Gestational weight gain was calculated as the weight measured after the 35 weeks gestation minus the pregestational weight. Gestational weight gain adequacy for each BMI category was classified as insufficient, adequate, or excessive according to the 2009 National Academy of Medicine recommendation ^(18)^. Preeclampsia was defined according to current recommendations ^(19)^. HbA1c was measured at booking, regardless of gestational age, and was collected at least once more ~ the 28th week. Assays were conducted with high-performance liquid chromatography (Variant II Turbo HbA1c; BioRad, Hercules, CA, USA) in line with the National Glycohemoglobin Standardization Program guidelines (http://www.ngsp.org/index. asp).

Birth weight categories were classified according to the World Health Organization chart ^(20)^ as small for gestational age (SGA, below the 10th percentile), large for gestational age (LGA, above the 90th percentile), and adequate (AGA, between the 10th and 90th percentiles). Macrosomia was defined as a birthweight ≥ 4,000 g. We defined congenital anomalies according to the Q chapter of the 10th International Classification of Diseases. Preterm birth refers to delivery before the 37+0 gestational weeks. Neonatal intensive care unit (NICU) admission and hypoglycemia diagnoses were accepted as determined by the neonatal assistant team. We excluded twin pregnancies and babies with genetic anomalies from the analyses of the neonatal outcomes.

Primary outcomes included maternal inpatient care for glycemic control, pre-eclampsia, NICU admission, and neonatal hypoglycemia. Gestational weight gain and 3rd-trimester HbA1c constituted the secondary outcomes.

### Statistical analysis

To explore the influence of the timing of overt diabetes diagnosis on pregnancy outcomes, we divided the sample into an early (up to 14 weeks gestation) and a late group (≥ 14 weeks) ^(5)^. We performed univariable analyses of the primary outcomes (maternal inpatient glycemic control, preeclampsia, neonatal hypoglycemia, and NICU admission) and secondary outcomes (total gestational weight gain and 3rd-trimester HbA1c) to identify relevant variables that were then entered into the multivariable analyses.

First, we calculated the crude risk for each outcome, testing early overt diabetes diagnosis against each of the selected outcomes. Then, we built multivariable models including potential confounders for early overt diagnosis and the variables found as relevant for each outcome by the univariable analyses. Baseline maternal characteristics (potential confounders) were included in the models only when a causal association was deemed probable. We tested the correlation between potentially related variables.

Continuous variables were described as mean and standard deviation or median and interquartile range, according to the profile of normal distribution analyzed by the Shapiro-Wilk test. Categorical variables were reported as absolute and relative frequencies. We used Student’s *t*-test or linear regression for continuous variables and the chi-square test for dichotomous variables to compare characteristics between groups.

Multivariable analyses were performed using Poisson regression with robust variances ^(21)^ for dichotomous outcomes, and the results were expressed as crude and adjusted relative risks (RR) and 95% confidence intervals (95% CI), p-value. We used linear models for continuous variables, and the results were presented as b coefficient (95% CI) and the corresponding p-value. We used ChatGPT 4o to adapt the English writing to that of a native speaker.

We followed the Strengthening the Reporting of Observational Studies in Epidemiology (STROBE) statement ^(22)^ to write the manuscript.

## RESULTS

Of the 646 participants with clinical characteristics of type 2 diabetes, 217 presented overt diabetes (33.6%, 95% CI 30.0%-37.4%); 127 (58.5%) were diagnosed before 14 gestational weeks and 90 (41.5%) after. In the **Supplementary Table 1**, we present the univariable analyses. Variables associated with maternal inpatient care included center of enrollment, gestational age, and weight gain at booking, and baseline HbA1c. Preeclampsia was associated with the center of enrollment, although two important clinical risk factors for this outcome, chronic hypertension and obesity, were included in the multivariable analyses. Maternal smoking, preeclampsia, gestational age at birth, male gender, and macrosomia were factors associated with neonatal hypoglycemia; smoking, preeclampsia, 3rd-trimester HbA1c, and gestational age at birth were associated with NICU admission. Gestational weight gain models included previous macrosomia, obesity, booking weight gain, and gestational age at delivery. The 3rd-trimester HbA1c models included previous macrosomia, booking weight gain, and booking HbA1c. Due to their clinical relevance as risk factors for the outcomes, these variables were included in the multivariable analyses along with late diagnosis of overt diabetes. Gestational age at booking was significant in univariable analyses of some outcomes. However, a moderate correlation (r = 0.645, p < 0.001) ^(23)^ between gestational age at booking and gestational age at overt diabetes diagnosis hindered the inclusion of the first variable in any multivariable analysis.

In **Supplementary Table 2**, we present the study power for the main outcomes using data from other studies and their prevalence in the current sample. Study power for different outcomes ranged from 23.2% (congenital anomaly) to 100% (NICU admission), considering a 5.0% significance level. Prevalence of outcomes in our study varied from 13.6% (congenital anomaly) to 38.6% (NICU admission). Although congenital anomalies exhibited an unexpected high frequency, we lacked an adequate sample size to evaluate the influence of early diagnosis on this outcome.

In **Table 1**, we summarize the participants’ baseline characteristics according to the timing of diagnosis, up to or after 14 gestational weeks. Fasting plasma glucose, alone or combined with HbA1c, was the most employed diagnostic tool for early diagnosis, whereas 2-h glucose was mostly used in the later period. Of the total sample, 94 women (78.3%) in the early group and 57 (65.5%) in the late group already presented a diagnostic fasting plasma glucose ≥ 126 mg/dL. Participants with early diagnosis more frequently had a family history of hypertension (56.7% vs. 36.7%, p = 0.006) and of previous hypertension (23.6% vs. 7.8%, p = 0.004), arrived earlier at the prenatal care facility (20.3 ± 7.7 weeks vs. 29.5 ± 5.5 weeks, p < 0.001) and required fewer hospital admissions for glycemic control during pregnancy (50.0% vs. 68.3%, p = 0.014). Total gestational weight gain was lower (6.5 kg ± 7.3 kg vs. 10.8 kg ± 10.5 kg, p = 0.002), and excessive weight gain was less frequent (26.1% vs. 44.3%, p = 0.004). They also reached a lower 3rd-trimester HbA1c (6.1% ± 0.9 % vs. 6.6% ± 1.2%, p = 0.02). Preeclampsia and neonatal (NICU admission and hypoglycemia) outcomes were similar between groups, and male gender was more common in early diagnosis pregnancies (67.6% vs. 46.8%, p = 0.007).

**Table 1:** Characteristics and outcomes of overt diabetes diagnosed before or after 14 gestational weeks in a cohort of pregnant Brazilian participants

Characteristic/outcome	Timing of overt diabetes diagnosis		p
	Up to 14 weeks	≥ 14 weeks	
	n = 127 (58.5)	n = 90 (41.5)	
Maternal			
Center			0.957
Center 1	59 (46.5)	43 (47.8)	
Center 2	68 (53.5)	47 (52.2)	
Maternal age (years)	32.3 ± 5.8	31.1 ± 6.5	0.153
White skin color	86 (67.7)	68 (75.6)	0.271
≤ 11 years of schooling	121 (95.3)	87 (96.7)	0.872
Smoking	16 (12.6)	10 (11.1)	0.904
Family history of diabetes	80 (63.0)	53 (58.9)	0.638
Previous gestational diabetes	46 (36.2)	24 (26.7)	0.182
Family history of hypertension	72 (56.7)	33 (36.7)	0.006
Chronic hypertension	30 (23.6)	7 (7.8)	0.004
Diabetes complications	2 (1.6)	0 (0.0)	0.635
Diabetes diagnosis by			<0.001
FPG	31 (24.4)	7 (7.8)	
HbA1c	14 (11.0)	5 (5.6)	
FPG + HbA1c	48 (37.8)	25 (27.8)	
2-h glucose	12 (9.4)	18 (20.0)	
2-h glucose + HbA1c	1 (0.8)	6 (6.7)	
FPG + 2-h glucose	7 (5.5)	8 (8.9)	
All	14 (11.0)	21 (23.3)	
FPG ≥ 126 mg/dL	94 (78.3)	57 (65.5)	0.059
	120	87	
Gestational age at diagnosis (weeks)	8.9 ± 3.0	22.4 ± 6.5	
Gestational age at booking (weeks)	20.3 ± 7.7	29.5 ± 5.5	<0.001
Folate use 1^st^ trimester	22 (17.3)	9 (10.0)	0.186
Obesity	95 (76.0)	56 (67.5)	0.233
	125	83	
Weight gain at booking (kg)	3.4 ± 5.6	7.9 ± 9.8	<0.001
	125	84	
HbA1c at booking (%)	7.0 ± 1.3	7.0 ± 1.3	0.692
	126	90	
HbA1c < 6.5% 1^st^ trimester	12 (22.6)	1 (100)	0.540
	53	1	
First HbA1c ≥ 7.0%	60 (47.6)	42 (46.7)	>0.999
	126	90	
Inpatient glycemic control	61 (50.0)	56 (68.3)	0.014
	122	82	
Gestational weight gain			
From booking to delivery (kg)	3.1 ± 4.3	2.5 ± 4.7	0.380
	121	80	
Total weight gain (kg)	6.5 ± 7.3	10.8 ± 10.5	0.002
Weight gain adequacy			0.005
Insufficient	51 (42.9)	33 (41.8)	
Adequate	37 (31.1)	11 (13.9)	
Excessive	31 (26.1)	35 (44.3)	
	119	79	
Insulin treatment during pregnancy	101 (82.1)	74 (83.1)	0.990
	123	89	
HbA1c 3rd-trimester (%)	6.1 ± 0.9	6.6 ± 1.0	0.005
	105	65	
HbA1c 3rd-trimester < 6.5%	73 (69.5)	30 (46.2)	0.004
	105	65	
Preeclampsia	38 (31.1)	28 (31.8)	>0.999
	122	88	
Neonatal			
Preterm birth	26 (23.4)	13 (16.5)	0.322
	111	79	
Male gender	75 (67.6)	37 (46.8)	0.007
	111	79	
Birth weight categories^*^			0.512
SGA	7 (6.3)	4 (5.1)	
AGA	64 (57.7)	40 (50.6)	
LGA	40 (36.0)	35 (44.3)	
	111	79	
Birth weight ≥ 4,000 g	18 (16.2)	12 (15.2)	>0.999
	111	79	
Congenital anomaly	14 (12.5)	6 (7.6)	0.395
	112	79	
NICU admission	42 (38.9)	28 (35.4)	0.743
	108	79	
Neonatal hypoglycemia	26 (24.1)	14 (17.7)	0.387
	108	79	
Neonatal death	1 (0.9)	0 (0.0)	>0.999
	108	79	

FPG: fasting plasma glucose; HbA1c: glycated hemoglobin; SGA: small for gestational age; AGA: adequate for gestational age; LGA: large for gestational age; NICU: neonatal intensive care admission.

* Birth weight categories classified according to the World Health Organization chart.

Data are presented as mean ± standard deviation or n (%).

Statistical tests: Student *t*-test for continuous variables and chi-square test for categorical variables.

In **Table 2**, we present the crude and adjusted risks for each outcome. The risk of maternal inpatient care for glycemic control increased by 37% (RR 1.37, 95% CI 1.08-1.72, n=204 women) with a late diagnosis. In multivariable analyses (n=195), this risk decreased to 29% (RR 1.29, 95% CI 1.02-1.63, p = 0.033) when adjusted for other associated factors (center of enrollment and baseline HbA1c).

**Table 2:** Risk factors for selected pregnancy outcomes in overt diabetes: comparing late with early diagnosis in a cohort of Brazilian participants

Maternal outcomes						
Inpatient glycemic control						
Risk factor	n	Crude RR (95% CI)	p	n	Adjusted RR (95% CI)	p
Late diagnosis	204	1.37 (1.08; 1.72)	0.008	195	1.29 (1.02; 1.63)	0.033
Center 2	204	1.44 (1.11; 1.86)	0.007		1.28 (1.01; 1.63)	0.046
Weight gain at booking	196	1.02 (1.01; 1.03)	<0.001		1.01 (1.00; 1.02)	0.096
HbA1c at booking	203	1.27 (1.17; 1.36)	<0.001		1.25 (1.16; 1.35)	<0.001
Preeclampsia						
Late diagnosis	210	1.02 (0.68; 1.53)	0.918	201	1.17 (0.78; 1.74)	0.446
Center	210	2.43 (1.50; 3.92)	<0.001		2.41 (1.48; 3.91)	<0.001
Chronic hypertension	210	1.60 (1.04; 2.46)	0.032		1.51 (0.96; 2.37)	0.076
Obesity	201	1.66 (0.97; 2.87)	0.067		1.65 (0.99; 2.76)	0.054
Total gestational weight gain						
		Crude b (95% CI)	p	n	Adjusted b (95% CI)	p
Late diagnosis	198	4.34 (1.85; 6.84)	0.001	184	-0.39 (-1.81; 1.04)	0.591
Previous macrosomia	198	3.11 (0.21; 6.02)	0.036		2.18 (0.64; 3.72)	0.006
Obesity	196	-6.77 (-9.24; -4.11)	<0.001		-1.98 (-3.53; -0.43)	0.012
Weight gain at booking	197	0.96 (0.88; 1.04)	<0.001		0.91 (0.82; 1.00)	<0.001
Maternal inpatient care	187	2.65 (0.08; 5.22)	0.043		-0.40 (-1.75; 0.95)	0.563
3rd-trimester Hba1c						
Late diagnosis	170	0.44 (0.15; 0.73)	0.003	169	0.29 (0.04; 0.55)	0.026
Previous macrosomia	170	0.49 (0.17; 0.80)	0.003		0.24 (-0.04; 0.52)	0.086
Weight gain at booking	169	0.03 (0.01; 0.05)	<0.001		0.02 (0.01; 0.04)	0.007
HbA1c at booking	170	0.36 (0.26; 0.46)	<0.001		0.33 (0.23; 0.42)	<0.001
Neonatal outcomes						
Hypoglycemia						
	n	Crude RR (95% CI)	p	n	Adjusted RR (95% CI)	p
Late diagnosis	187	0.74 (0.41; 1.32)	0.302	186	0.93 (0.54; 1.61)	0.788
Smoking	187	2.46 (1.42; 4.27)	0.001		1.86 (1.08; 3.18)	0.025
Preeclampsia	186	1.89 (1.09; 3.27)	0.023		1.59 (0.94; 2.69)	0.086
Gestational age at delivery	187	0.85 (0.75; 0.96)	0.011		0.86 (0.75; 0.99)	0.032
Male gender	187	2.31 (1.17; 4.56)	0.016		1.88 (0.95; 3.73)	0.071
Macrosomia	187	1.99 (1.12; 3.52)	0.019		1.99 (1.16; 3.42)	0.013
NICU admission						
Late diagnosis	187	0.91 (0.62; 1.33)	0.632	152	0.86 (0.54; 1.35)	0.497
Smoking	187	2.08 (1.47; 2.94)	<0.001		2.22 (1.47; 3.36)	<0.001
Preeclampsia	186	1.70 (1.18; 2.44)	0.004		1.56 (1.04; 2.35)	0.033
3rd-trimester HbA1c	152	1.29 (1.06; 1.56)	0.010		1.39 (1.14; 1.68)	0.001
Gestational age at delivery	187	0.86 (0.81; 0.92)	<0.001		0.85 (0.77; 0.93)	<0.001

RR: relative risk; CI: confidence interval; HbA1c: glycated hemoglobin.

Data are presented as crude and adjusted relative risk (95% CI) or b coefficient (95% CI).

Statistics tests: Poisson’s regression with robust variances, linear regression.

In the preeclampsia models (n = 201), the center of enrollment was associated with a greater than two-fold risk (RR: 2.41, 95% CI 1.48-3.91, p < 0.001). Neither early diagnosis nor other relevant risk factors (chronic hypertension and obesity) were significant in the multivariable analysis.

Early diagnosis showed no effects on the neonatal outcomes. Several expected risk factors related to hypoglycemia (n = 186 women) were associated in adjusted models: gestational age at delivery (RR 0.86, 95% CI 0.75-0.99, p = 0.032), macrosomia (RR 1.99, 95% CI 1.16-3.43, p = 0.013), and smoking (RR 1.86, 95% CI 1.08-3.18, p = 0.025). Smoking (RR 2.22, 95% CI 1.47-3.36, p < 0.001) and a higher maternal 3rd-trimester HbA1c (RR 1.39, 95% CI 1.14-1.68, p < 0.001) were associated with an increased risk for NICU admission (n = 152 neonates), whereas a higher gestational age at delivery (RR 0.85, 95% CI 0.77-0.93, p < 0.001) was protective.

Gestational weight gain and 3rd-trimester HbA1c were associated with an early diagnosis of diabetes in non-adjusted models. In adjusted models, a late diagnosis was associated with an increased 3rd-trimester HbA1c value (b = 0.29, 95% CI 0.04-0.55, p = 0.026).

## DISCUSSION

In this cohort of 217 women with diabetes first diagnosed during pregnancy, mostly before 14 weeks gestation, early diagnosis did not improve main pregnancy outcomes, even when adjusted for clinically relevant variables. A late diagnosis was associated with a higher risk of maternal inpatient care for glycemic control and a higher 3rd-trimester HbA1c level.

The clinical implications of the timing of overt diabetes diagnosis remain insufficiently investigated. In our cohort, the physiological alterations of pregnancy, further enhanced by an already increased insulin resistance due to a previous undiagnosed hyperglycemia (plus the effects of obesity), could explain the differences observed in the comparisons between the first and later trimesters of pregnancy. We posit that insulin sensitivity was affected earlier in these pregnancies due to pregestational insulin resistance associated with obesity. Among studies with more than 100 participants, one reported no significant differences in pregnancy outcomes when comparing diagnoses made before and after the 20th gestational week ^(7)^. When applying this cutoff to our sample, baseline maternal characteristics and pregnancy outcomes were similar, limiting further relevant analyses. Metabolic changes related to placental secretion of hormones that compete with insulin are enhanced from the second trimester onwards, partially justifying the similarity found between the groups. Moreover, the behavior of metabolic changes during pregnancy can differ between overt diabetes and GDM, as insulin resistance is probably more pronounced at the beginning of pregnancy in overt diabetes ^(6)^.

Other studies on the timing of overt diabetes diagnosis are available. In a recent study from China, the authors found an association between early diagnosis and increased risks of macrosomia and large-for-gestational-age (LGA) neonates ^(13)^. Smaller studies have suggested increased risk of LGA ^(12)^ and postpartum diabetes associated with first-trimester overt diabetes ^(4)^. Our findings revealed an association between early diagnosis of overt diabetes and some maternal benefits, such as reduced hospitalization for glycemic control; however, this improvement did not translate into better maternal and neonatal outcomes. Delayed access to specialized prenatal care in Brazil can partially explain this finding. Although some initial treatment steps could have been implemented in the primary care setting, these measures are not standardized in the general prenatal care. Even women diagnosed in the first trimester arrived at the specialized care after ~20 gestational weeks, despite diabetes in pregnancy being a referral priority in the Brazilian public health system ^(24)^. In a study of 319 women, only ~33% reported arrival before 15 days after referral to a specialized unit; another 27% arrived less than 30 days before, and almost 20% never sought the service ^(25)^. A multifactorial issue, one of the reasons for such delays includes the public health system’s prioritized attention on more prevalent infectious diseases. Paucity of specialized teams and the socioeconomic, educational, and cultural characteristics of patients also contribute ^(24)^. Although women diagnosed in the first trimester arrived at specialists around mid-pregnancy, they had received nutritional counseling and medications, including insulin, in the primary setting, gaining less weight (~3.0 kg) compared with those arriving later.

Most outcomes evaluated showed no relation with early diagnosis of overt diabetes, but did correlate with other clinically relevant variables. Although chronic hypertension, a well-known risk factor for preeclampsia ^(19)^, was more prevalent (~ three times) among women diagnosed with overt diabetes during the first trimester, it showed no significant association with preeclampsia after adjustments. This finding suggests that the presence of chronic hypertension in early pregnancy prompted clinicians and obstetricians to screen for diabetes earlier. Chronic hypertension has been inadequately reported in studies on overt diabetes (≥ 100 participants) - ~4.0% in one study ^(26)^, ~9.0% in another ^(27)^, compared to ~17.0% in our cohort - limiting our evaluation of chronic hypertension as a risk factor for overt diabetes.

As expected, neonatal hypoglycemia showed a protective association with a higher gestational age at delivery, whereas neonatal macrosomia was associated with increased risk of this outcome ^(28)^.

NICU admission was independently related to higher gestational age at delivery (a protective role) and to maternal third-trimester glycemia, highlighting the role of late-pregnancy glycemic control in reducing neonatal morbidity, as found in other types of diabetes in pregnancy ^(29)^. Preeclampsia and smoking were other independent risk factors, possibly related to placental function.

Overt diabetes remains poorly characterized in the literature. Many studies of diabetes in pregnancy excluded this group ^(30,31)^. The reported prevalence of overt diabetes varies widely, ranging from 0.23% among US women with “gestational diabetes progressing to type 2 diabetes” ^(32)^ to 0.4% in a French national cohort ^(10)^ and up to 21.7% ^(7)^ in a high-risk population. Whether earlier diagnosis and intervention translate into improved outcomes is unclear. No randomized controlled trials have addressed this question so far. One observational study reported comparable outcomes in women with well-controlled overt diabetes and those with gestational diabetes or normoglycemic pregnancies ^(33)^.

Lack of postpartum follow-up data hindered evaluating persistent diabetes in our cohort. Previous studies reported postpartum diabetes rates between 19.5% ^(7)^ and 70.7% ^(4)^. In a meta-analysis, the risk of diabetes in women presenting overt diabetes was 6-fold compared with women with previous GDM and 25-fold compared with women without hyperglycemia ^(3)^. Many women in our sample probably had undiagnosed type 2 diabetes, as most of them presented severe hyperglycemia already at diagnosis: more than 75% of those diagnosed in the first trimester had a fasting plasma glucose ≥ 126 mg/dL, the current diagnostic cut-off point for diabetes in non-pregnant adults. Moreover, the traditional cut point for diabetes control, a HbA1c of 7.0%, was found for half of the sample. Only two participants presented diabetes complications, but their presence ratifies the existence of an unknown hyperglycemia before pregnancy. The small sample size did not allow further sensitivity analyses according to hyperglycemia severity.

As strengths of our work, this is one of the first studies with over 100 participants to assess the impact of an early diagnosis of overt diabetes on pregnancy outcomes. Early diagnosis was correlated with some improved maternal parameters during pregnancy, such as less total gestational weight gain in women with obesity and a lower 3rd-trimester HbA1c, but not with better pregnancy outcomes. This may be due to the homogeneous sample enrolled, as age and BMI were quite similar between the groups.

A strong limitation of our study was the relatively small sample size which may have reduced the power to detect differences in rarer outcomes, such as perinatal death and congenital anomalies. Study power for the main outcomes was high for neonatal hypoglycemia and NICU admission (90-100%) and moderate for preeclampsia (~76%). Some results with a null association (when it was expected to occur) may reflect type II error, related to the study power. Another limitation is the influence of the study center on the outcomes, which reflects, first, the retrospective and secondary collection of information, and second, the confidence in the diagnostic definitions made by the medical team caring for the participants, likely due to different patterns of assistance. The generalization of the results is limited due to the exclusive inclusion of women who used the public health system, mostly having a lower income profile associated with a lower educational level (only 4.1% had over 11 years of schooling). Data on high-risk prenatal care in the private setting in Brazil (as accessed in PubMed and Embase), which could yield different results associated with higher educational and socioeconomic backgrounds, is lacking. Nevertheless, the cohort is likely representative of Latina women with overt diabetes: they presented higher rates for family history of diabetes (~60% compared with <23% in one Chinese cohort ^(13)^ and ~57% in a New Zealand cohort ^(7)^), and obesity was common (73% compared with < 42% in a Chinese cohort ^(13)^). Finally, lack of postpartum data limits firm conclusions on diabetes persistence, a key factor for a definitive classification of hyperglycemia.

In conclusion, in the present cohort of women with overt diabetes cared for in the public health system, early diagnosis was associated with better maternal control, but this did not translate into improved clinically relevant pregnancy outcomes. Our findings underscore the need for prospective trials to determine a timely diagnosis and intervention, helping to improve pregnancy outcomes in these women.

## Data Availability

the datasets used and/or analyzed during the current study are available from the corresponding author on reasonable request. Inquiries can be directed to the corresponding author (areichelt@hcpa.edu.br) or to Vania Hirakata (vhirakata@hcpa.edu.br). Preliminary results were presented as a poster at the XXV Congress of the Brazilian Society of Diabetes (*Sociedade Brasileira de Diabetes*), held in Rio de Janeiro, October 29 to 31, 2025.

## SUPPLEMENTARY MATERIAL

**Supplementary Table 1:** Overt diabetes in pregnancy: univariable analyses by outcome

Maternal						
Characteristic/outcome	Inpatient glycemic control		p	Preeclampsia		p
	no	yes		no	yes	
	n = 87	n = 117		n = 144	n = 66	
Late diagnosis	26 (29.9)	56 (47.9)	0.014	60 (41.7)	28 (42.4)	>0.999
Center			0.006			<0.001
Center 1	48 (55.2)	41 (35.0)		79 (54.9)	17 (25.8)	
Center 2	39 (44.8)	76 (65.0)		65 (45.1)	49 (74.2)	
Maternal age	32.1 ± 5.8	31.8 ± 6.5	0.760	32.1 ± 6.0	31.3 ± 6.1	0.380
White skin color	61 (70.1)	80 (68.4)	0.910	103 (71.5)	44 (66.7)	0.581
≤ 11 years in school	81 (93.1)	115 (98.3)	0.128	138 (95.8)	65 (98.5)	0.562
Smoking	11 (12.6)	12 (10.3)	0.757	17 (11.8)	8 (12.1)	>0.999
Family history of diabetes	52 (59.8)	78 (66.7)	0.386	87 (60.4)	43 (65.2)	0.615
Previous gestational diabetes	29 (33.3)	38 (32.5)	>0.999	50 (34.7)	18 (27.3)	0.362
Family history of hypertension	44 (50.6)	60 (51.3)	>0.999	65 (45.1)	38 (57.6)	0.127
Chronic hypertension	16 (18.4)	19 (16.2)	0.829	19 (13.2)	16 (24.2)	0.073
Diabetes complications	1 (1.1)	1 (0.9)	>0.999	1 (0.7)	1 (1.5)	>0.999
FPG ≥ 126 mg/dL	61 (70.9)	86 (78.9)	0.265	98 (70.0)	49 (80.3)	0.178
	86	109		140	61	
Gestational age at booking	22.1 ± 7.7	25.4 ± 8.4	0.005	23.7 ± 8.0	25.1 ± 8.4	0.262
Obesity	59 (72.0)	81 (71.7)	>0.999	93 (68.4)	53 (81.5)	0.074
	82	113		136	65	
Weight gain at booking (kg)	3.5 ± 6.7	6.5 ± 8.5	0.010	5.0 ± 7.1	5.8 ± 9.3	0.489
	83	113		138	64	
HbA1c at booking	6.5 ± 1.1	7.5 ± 1.2	<0.001	7.0 ± 1.4	7.1 ± 1.1	0.515
	86	117		143	66	
Weight gain adequacy			0.061			0.082
Insufficient	41 (51.9)	38 (35.2)		65 (47.1)	18 (31.0)	
Adequate	15 (19.0)	32 (29.6)		33 (23.9)	15 (25.9)	
Excessive	23 (29.1)	38 (35.2)		40 (29.0)	25 (43.1)	
	79	108		138	58	
3rd-trimester HbA1c	6.0 ± 0.8	6.5 ± 1.0	0.002	6.3 ± 1.0	6.4 ± 0.8	0.466
	67	95		119	49	
Characteristic/outcome	Total gestational weight gain		p	3rd-trimester HbA1c		p
	n	b (95% CI)		n	b (95% CI)	
Late diagnosis	198	4.34 (1.85; 6.84)	0.001	170	0.44 (0.15; 0.73)	0.003
Center	198	0.04 (-2.49; 2.57)	0.975	170	0.11 (-0.18; 0.40)	0.462
Maternal age	198	-0.08 (-0.28; 0.13)	0.471	170	-0.02 (-0.04; 0.01)	0.208
White skin color	198	-1.33 (-4.08; 1.41)	0.340	170	-0.03 (-0.35; 0.29)	0.857
≤ 11 years in school	198	-0.03 (-0.99; 0.94)	0.951	170	-0.08 (-0.19; 0.04)	0.195
Smoking	198	-0.91 (-4.69; 2.88)	0.638	170	-0.19 (-0.66; 0.28)	0.431
Family history of diabetes	198	-0.01(-2.61; 2.59)	0.993	170	0.20 (-0.10; 0.49)	0.195
Previous gestational diabetes	198	-1.08 (-3.74; 1.57)	0.422	170	0.16 (-0.14; 0.46)	0.300
Previous macrosomia	198	3.11 (0.21; 6.02)	0.036	170	0.49 (0.17; 0.80)	0.003
Characteristic/outcome	Total gestational weight gain		p	3rd-trimester HbA1c		p
	n	b (95% CI)		n	b (95% CI)	
Chronic hypertension	198	-0.25 (-3.63; 3.12)	0.884	170	-0.13 (-0.52; 0.26)	0.515
Diabetes complications	198	-1.01 (-18.75; 16.74)	0.911	170	-0.29 (-1.63; 1.04)	0.666
Number of pregnancies	198	0.40 (-0.39; 1.18)	0.321	170	0.01 (-0.08; 0.11)	0.782
Pregestational BMI	197	-0.45 (-0.59; -0.31)	<0.001	168	0.00 (-0.02; 0.01)	0.626
Obesity	196	-6.77 (-9.42; -4.11)	<0.001	167	-0.05 (-0.37; 0.27)	0.766
Gestational age at booking	198	0.30 (0.15; 0.45)	<0.001	170	0.03 (0.01; 0.05)	0.001
Weight gain at booking	197	0.96 (0.88; 1.04)	<0.001	169	0.03 (0.01; 0.05)	<0.001
HbA1c at booking	197	0.94 (-0.04-1.92)	0.060	170	0.36 (0.26; 0.46)	<0.001
Maternal inpatient care	187	2.65 (0.08; 5.22)	0.043	162	0.46 (0.17; 0.76)	0.002
3rd-trimester HbA1c	162	3.01 (1.59; 4.43)	<0.001	---		
Total gestational weight gain	---			162	0.03 (0.02; 0.05)	<0.001
Gestational age at delivery	189	0.00 (-0.44; 0.45)	0.986	162	0.03 (-0.04; 0.09)	0.411
Neonatal						
Characteristic/outcome	Hypoglycemia		p	NICU admission		p
	no	yes		no	yes	
	n = 147	n = 40		n = 117	n = 70	
Late diagnosis	65 (44.2)	14 (35.0)	0.387	51 (43.6)	28 (40.0)	0.743
Center			>0.999			0.509
Center 1	70 (47.6)	19 (47.5)		53 (45.3)	36 (51.4)	
Center 2	77 (52.4)	21 (52.5)		64 (54.7)	34 (48.6)	
Maternal age	32.1 ± 6.4	30.6 ± 5.1	0.162	32.5 ± 6.4	30.7 ± 5.6	0.063
White skin color	103 (70.1)	26 (65.0)	0.673	81 (69.2)	48 (68.6)	>0.999
≤ 11 years in school	142 (96.6)	39 (97.5)	>0.999	113 (96.6)	68 (97.1)	>0.999
Maternal smoking	14 (9.5)	11 (27.5)	0.007	8 (6.8)	17 (24.3)	0.002
Obesity	107 (76.4)	24 (63.2)	0.150	84 (75.0)	47 (71.2)	0.706
Preeclampsia	40 (27.2)	18 (46.2)	0.038	28 (23.9)	30 (43.5)	0.009
Total gestational weight gain	7.5 ± 9.0	10.4 ± 7.2	0.062	7.3 ± 8.7	9.5 ± 8.5	0.099
	139	38		112	65	
3rd-trimester HbA1c	6.2 ± 1.0	6.5 ± 0.8	0.165	6.2 ± 0.9	6.6 ± 1.1	0.012
	122	30		101	51	
Gestational age at birth	37.9 ± 1.6	37.2 ± 2.0	0.027	38.1 ± 1.2	37.2 ± 2.2	0.004
Preterm birth	20 (13.6)	18 (45.0)	<0.001	11 (9.4)	27 (38.6)	<0.001
Male gender	81 (55.1)	31 (77.5)	0.017	64 (54.7)	48 (68.6)	0.086
Macrosomia (≥ 4,000 g)	19 (12.9)	11 (27.5)	0.047	16 (13.7)	14 (20.0)	0.350
Birth weight categories^*^			0.085			0.126
SGA	9 (6.1)	1 (2.5)		9 (7.7)	1 (1.4)	
AGA	85 (57.8)	17 (42.5)		65 (55.6)	37 (52.9)	
LGA	53 (36.1)	22 (55.0)		43 (36.8)	32 (45.7)	

* Birth weight categories classified according to the World Health Organization chart.

FPG: fasting plasma glucose; HbA1c: glycated hemoglobin; BMI body mass index; NICU: neonatal intensive care unit SGA: small for gestational age; AGA: adequate for gestational age; LGA: large for gestational age.

Data are presented as n (%) or mean ± standard deviation unless otherwise highlighted.

**Supplementary Table 2:** Sample size and power according to the main pregnancy outcomes

Outcome	Sample size	RR	% (literature)	Power	RR	% (this study)
Preeclampsia	120	2.0	28.2	75.8	2.0	30.1
Hypoglycemia	178	2.0	22.0	90.8	2.0	23.8
NICU	220	2.0	18.9	100.0	2.0	38.6
Congenital anomaly	880	2.0	5.9	23.3	2.0	13.6

RR: relative risk; %: frequency.

All parameters were calculated using the PSS Health online version ^(17)^, with a power of 80% and a level of significance of 5%, plus 10% for losses.

Since the frequency of outcomes varied in the literature, we estimated the mean prevalence of hypoglycemia and NICU. The prevalence of preeclampsia and congenital anomalies was based on the study of Jovanovič and cols. ^(32)^.

The relative risk of 2.0 or all calculations was arbitrarily set based on the study of Jayasinghe and cols. ^(12)^.

## References

[1] Diagnostic criteria and classification of hyperglycaemia first
detected in pregnancy: a World Health Organization Guideline (2014). Diabetes Res Clin Pract.

[2] Simmons D. (2021). Paradigm Shifts in the Management of Diabetes in Pregnancy: The
Importance of Type 2 Diabetes and Early Hyperglycemia in Pregnancy: The 2020
Norbert Freinkel Award Lecture. Diabetes Care.

[3] Pal A, Rawat D, Sadananda S, Goyal A, Haldar P, Garg D (2025). Pregnancy and postpartum cardiometabolic outcomes among women
with overt diabetes compared to women with normoglycaemia, gestational
diabetes and pre-existing diabetes: A systematic review and
meta-analysis. Diabetes Obes Metab.

[4] Gupta Y, Goyal A, Ambekar S, Kalaivani M, Bhatla N, Tandon N. (2024). Cardiometabolic profile of women with a history of overt diabetes
compared to gestational diabetes and normoglycemia in index pregnancy:
Results from CHIP-F study. J Diabetes.

[5] Lain KY, Catalano PM. (2007). Metabolic changes in pregnancy. Clin Obstet Gynecol.

[6] Scairati R, McEvoy RP, Newman C, Dunne FP. (2025). Outcomes of pregnancies complicated by type 2
diabetes. Best Pract Res Clin Endocrinol Metab.

[7] Immanuel J, Eagleton C, Baker J, Simmons D. (2021). Pregnancy outcomes among multi-ethnic women with different
degrees of hyperglycaemia during pregnancy in an urban New Zealand
population and their association with postnatal HbA1c uptake. Aust N Z J Obstet Gynaecol.

[8] Brasil (2024). Caderneta da gestante.

[9] Oppermann ML, Campos MA, Hirakata VN, Reichelt AJ. (2022). Overt diabetes imposes a comparable burden on outcomes as
pregestational diabetes: a cohort study. Diabetol Metab Syndr.

[10] Regnault N, Lebreton E, Tang L, Fosse-Edorh S, Barry Y, Olié V (2024). Maternal and neonatal outcomes according to the timing of
diagnosis of hyperglycaemia in pregnancy: a nationwide cross-sectional study
of 695,912 deliveries in France in 2018. Diabetologia.

[11] Bhattacharya S, Nagendra L, Dutta D, Kamrul-Hasan ABM. (2025). Treatment Versus Observation in Early Gestational Diabetes
Mellitus: A Systematic Review and Meta-analysis of Randomized Controlled
Trials. J Clin Endocrinol Metab.

[12] Jayasinghe IU, Koralegedara IS, Agampodi SB. (2022). Early pregnancy hyperglycaemia as a significant predictor of
large for gestational age neonates. Acta Diabetol.

[13] Shu X, Juan J, Kang X, Yao M, Chen X, Wei Z, Kong L, Chen H, Cui S, Gao F, Zhu P, Yan J, Xu X, Zhang L, Wang Y, Mi Y, Yang H. (2025). Comparative analysis of perinatal outcomes in pregnant women with
pregestational diabetes mellitus based on diagnostic timing. Sci Rep.

[14] Mañé L, Flores-Le Roux JA, Benaiges D, Chillarón JJ, Prados M, Pedro-Botet J (2019). Impact of overt diabetes diagnosed in pregnancy in a multi-ethnic
cohort in Spain. Gynecol Endocrinol.

[15] Milln J, Nakabuye B, Natamba BK, Sekitoleko I, Mubiru M, Namara AA (2021). Antenatal management and maternal/fetal outcomes associated with
hyperglycaemia in pregnancy (HIP) in Uganda; a prospective cohort
study. BMC Pregnancy Childbirth.

[16] American Diabetes Association Professional Practice
Committee (2025). 2. Diagnosis and Classification of Diabetes: Standards of Care in
Diabetes-2025. Diabetes Care.

[17] Borges RB, Mancuso ACB, Camey SA, Leotti VB, Hirakata VN, Azambuja GS (2021). Power and Sample Size for Health Researchers: uma ferramenta para
cálculo de tamanho amostral e poder do teste voltado a pesquisadores
da área da saúde. Clin Biomed Res.

[18] Rasmussen KM, Yaktine AL, Institute of Medicine (US) and National Research Council (US)
Committee to Reexamine IOM Pregnancy Weight Guidelines (2009). Weight gain during pregnancy: reexamining the guidelines.

[19] Magee LA, Brown MA, Hall DR, Gupte S, Hennessy A, Karumanchi SA (2022). The 2021 International Society for the Study of Hypertension in
Pregnancy classification, diagnosis & management recommendations for
international practice. Pregnancy Hypertens.

[20] Villar J, Cheikh Ismail L, Victora CG, Ohuma EO, Bertino E, Altman DG, Lambert A, Papageorghiou AT, Carvalho M, Jaffer YA, Gravett MG, Purwar M, Frederick IO, Noble AJ, Pang R, Barros FC, Chumlea C, Bhutta ZA, Kennedy SH, International Fetal and Newborn Growth Consortium for the 21st
Century (INTERGROWTH-21st) (2014). International standards for newborn weight, length, and head
circumference by gestational age and sex: the Newborn Cross-Sectional Study
of the INTERGROWTH-21st Project. Lancet.

[21] Barros AJ, Hirakata VN. (2003). Alternatives for logistic regression in cross-sectional studies:
an empirical comparison of models that directly estimate the prevalence
ratio. BMC Med Res Methodol.

[22] von Elm E, Altman DG, Egger M, Pocock SJ, Gøtzsche PC, Vandenbroucke JP, STROBE Initiative (2008). The Strengthening the Reporting of Observational Studies in
Epidemiology (STROBE) statement: guidelines for reporting observational
studies. J Clin Epidemiol.

[23] Schober P, Boer C, Schwarte LA. (2018). Correlation Coefficients: Appropriate Use and
Interpretation. Anesth Analg.

[24] Universidade Federal do Rio Grande do Sul (2024). Faculdade de Medicina, TelessaúdeRS-UFRGS. Protocolos de
regulação ambulatorial: obstetrícia (pré-natal
de alto risco) [recurso eletrônico].

[25] Fontana Medeiros F, Santos I, Franchi J, Caldeira S, Ferrari R, Cardelli A. (2022). Tempo de espera ao acesso ambulatorial especializado no
pré-natal de alto risco: estudo de método
misto. Res Soc Dev.

[26] Immanuel J, Simmons D. (2017). Screening and Treatment for Early-Onset Gestational Diabetes
Mellitus: a Systematic Review and Meta-analysis. Curr Diab Rep.

[27] Sugiyama T, Saito M, Nishigori H, Nagase S, Yaegashi N, Sagawa N, Japan Diabetes and Pregnancy Study Group (2014). Comparison of pregnancy outcomes between women with gestational
diabetes and overt diabetes first diagnosed in pregnancy: a retrospective
multi-institutional study in Japan. Diabetes Res Clin Pract.

[28] D’Souza R, Horyn I, Pavalagantharajah S, Zaffar N, Jacob CE. (2019). Maternal body mass index and pregnancy outcomes: a systematic
review and metaanalysis. Am J Obstet Gynecol MFM.

[29] American Diabetes Association Professional Practice
Committee (2025). 15. Management of Diabetes in Pregnancy: Standards of Care in
Diabetes-2025. Diabetes Care.

[30] Newman C, Egan AM, Ahern T, Al-Kiyumi M, Bacon S, Bahaeldein E (2022). Retrospective national cohort study of pregnancy outcomes for
women with type 1 and type 2 diabetes mellitus in Republic of
Ireland. Diabetes Res Clin Pract.

[31] Murphy HR, Howgate C, O’Keefe J, Myers J, Morgan M, Coleman MA, National Pregnancy in Diabetes (NPID) advisory group (2021). Characteristics and outcomes of pregnant women with type 1 or
type 2 diabetes: a 5-year national population-based cohort
study. Lancet Diabetes Endocrinol.

[32] Jovanovič L, Liang Y, Weng W, Hamilton M, Chen L, Wintfeld N. (2015). Trends in the incidence of diabetes, its clinical sequelae, and
associated costs in pregnancy. Diabetes Metab Res Rev.

[33] Park S, Kim SH. (2015). Women with rigorously managed overt diabetes during pregnancy do
not experience adverse infant outcomes but do remain at serious risk of
postpartum diabetes. Endocr J.

